# Recent Trends in Fluorescent Organic Materials for Latent Fingerprint Imaging

**DOI:** 10.3389/fchem.2020.594864

**Published:** 2020-11-09

**Authors:** Jie Lian, Fanda Meng, Wei Wang, Zhitao Zhang

**Affiliations:** ^1^College of Criminal Investigation, People's Public Security University of China, Beijing, China; ^2^Institute of Basic Medicine, Shandong First Medical University & Shandong Academy of Medical Sciences, Shandong, China

**Keywords:** fluorescent imaging, latent fingerprints development, conjugated oligomer, high contrast, sensitive imaging, fluorescent organic small molecules

## Abstract

Fingerprints are an important kind of material evidence with the key function in personal identification, which are unique and life-long to everyone. Latent (invisible) fingerprints are common at the crime scene, needing to be visualized with proper methods in order to identify sources of the fingerprints in routine forensic practice. Fluorescent imaging of latent fingerprints has the advantage of high contrast, sensitivity, selectivity, and less dependency on instruments. Taking the environment and users' safety into consideration, organic materials for fluorescent imaging of latent fingerprints are reviewed mainly in recent 5 years. New strategies of fingerprint reagents and improved performances established for fingerprint development based on fluorescent organic materials are discussed in the view of forensic practice. In addition, we briefly highlight current challenges of recent fluorescent imaging works based on organic materials for the latent fingerprints development in forensic practice.

## Introduction

Fingerprints have been one of the most important gold biometric features for personal identification in the forensic science field for more than 100 years, because of the uniqueness and lasting consistency over the lifetime. A fingerprint is an impression of the pattern of ridges (raised) and furrows (recessed) formed when a finger makes contact with a surface. Most fingerprints are invisible to the naked eye at the crime scene, also known as latent fingerprints (LFPs). LFPs could be exploited as a potential key evidence in countless cases of serious crime, requiring a special development process in order to visualize them by the police or other authorities. To identify individuals with fingerprints, there are three levels of fingerprint recognition. The first-level features are macro details mainly used for the pattern classification, not distinctive enough to recognize fingerprint. The second-level features (minutia points) are distinctive and stable, widely used for distinguishing the uniqueness of fingerprint, while the third-level features (pores and ridge contours) are the dimension attributes of the ridges to provide more accurate and robust details for accurate fingerprint recognition. Nowadays, many methods exist as standard methods for the development of the LFPs on common substrates in routine forensic practice (Ezhilmaran and Adhiyaman, 2017; Lennard, 2020), but there are still some situations that it is difficult or impossible to recover LFPs for forensic investigators. The ongoing research is being directed at improved sensitivity, universality, convenience, and efficiency via the optimization of existing methods or new approaches, such as spectroscopy, mass spectrometry, immuno-labeling, and nanoparticles based approaches (Nakamura et al., 2015; Zhao et al., 2016; Figueroa et al., 2017; O'Neill et al., 2018; Kolhatkar et al., 2019; Bodelón and Pastoriza-Santos, 2020; Li et al., 2020).

As latent fingerprint may exist everywhere at the crime scene and the visualization technology requires high quality and speed to identify individuals in forensic sciences, the visible detection of the LFPs on different surfaces have been explored through a variety of spectroscopy methods, also called LFPs imaging, visualization, detection, or developing methods. The most commonly used procedures for developing LFPs are powder-dusting and spraying methods in both forensic practice and research field, and the developed images are stored as evidences, so colored dusting powders have attracted much attention from related research community (Cadd et al., 2015; Friesen, 2015; Huynh and Halámek, 2016). The powder-dusting method relies on the adherence of powder particles to the fingerprint deposits, forming patterns of ridges, and furrows for fingerprint identification. The fingerprint powders are categorized into metal powders, magnetic powders, and colored or fluorescent powders. Among them, colored or fluorescent powders are usually applied after cyanoacrylate fuming, and most of fluorescent powders are fluorescent organic small molecules, widely used on different surfaces under light irradiation for the high contrast with the surface background. Although organic small molecules are successfully used for LFPs imaging, fluorescent organic small molecules need to be applied with other matrix to improve their low biocompatibility, low water solubility, and toxicity to get better performance of LFP fluorescent imaging, making this process more convenient (Sodhi and Kaur, 2000; Chen et al., 2009; Yuan et al., 2018; Bhagat et al., 2020). The research of fluorescent materials also has emerged, showing them to be new agents for developing LFPs, due to their unique optical and chemical properties with higher fluorescent intensity, better fluorescence stability, and higher contrast. Many reports studied widely on heavy metal quantum dots, rare earth nanoparticles, and noble metal nanoparticles for fluorescent imaging of LFPs, but their raw materials are limited, not easy to get, and potentially dangerous to the environment and users (Liu et al., 2010; Niu et al., 2015; Li et al., 2017; Bharat et al., 2018; Costa et al., 2020a; Ghubish et al., 2020; Gouiaa et al., 2020; Kumar et al., 2020; Park et al., 2020). As a result, researchers now turn back to fluorescent organic materials and utilize various synthesis strategies and reagent compositions to seek environmentally friendly, biocompatible, water-soluble, and low toxic fingerprint developing materials on various substrates.

Consequently, the mini-review intends to highlight recent advances of LFP development techniques by using various fluorescent organic materials mainly in the recent 5 years. The following sections are categorized into two parts according to the chemical structures of fluorescent organic materials, which encompasses fluorescent organic small molecule-based materials and fluorescent polymer-based materials. The intention of this mini-review is not to provide an exhaustive literature survey but to showcase the possibility to apply for LFP development and the trend of the fluorescent organic material synthesis and modification strategies, and the organic materials are now not used alone as the LFP detection reagents. Finally, the conclusions and future perspectives section is given to discuss the existing challenges and future perspectives for fluorescent organic material-based LFP imaging methods. Without a doubt, this emerging field contains numerous possibilities, and the further development for LFP imaging will be further advanced with new types of organic materials.

## Fluorescent Organic Small Molecule-Based Materials for LFP Imaging

Common traditional fluorescent organic small molecules, such as fluorescein, rhodamine 6 G, rhodamine B, brilliant Blue G-250, curcumin, and benzazole dyes, have already been used as LFP reagents. However, fluorescent organic small molecules could only be used on limited situations, due to the low affinity to fingerprint residues and other physical and chemical properties. To improve the interaction between organic small molecules and LFPs, the modified organic small molecules with different functional groups and various matrices including non-toxic or low-toxic nanomaterials have been developed as LFP imaging reagents. The matrix used by fluorescent organic small molecules for LFP imaging acts as a carrier, bioaffinity improver, and fluorescence developer (Barros et al., 2017; Brunelle et al., 2017; Zhang et al., 2017; Alsolmy et al., 2018; Uppal et al., 2018; Duan et al., 2019). On the other hand, aggregation-induced emission (AIE) exhibits remarkable bright fluorescence in aggregate or solid state (Mei et al., 2015; Zhao et al., 2017; Chen et al., 2018), and AIE-active molecules solely have been used for LFP bioimaging with high contrast and short developing time (Li et al., 2012; Xu et al., 2014; Suresh et al., 2018).

### Fluorescent Organic Small Molecules With Different Matrices for LFP Imaging

Fluorescent organic small molecules for LFP imaging adsorbed to natural matrices, such as starch and various types of clays including montmorillonite and diatomite, validated as excellent LFP powders on various surfaces (Chen et al., 2009; Yuan et al., 2018; Yadav, 2019). Three synthesized organic small molecules were made into the formulation of the proposed fluorescent developers based on starch, reducing any risk of inherent toxicity. The influence of fingerprint age (up to 30 days) and sequential deposition (6 times) were studied on porous and non-porous substrates and received satisfying results. These fluorescent powders were demonstrated to be efficient and promising for detecting fresh and non-fresh fingerprints on porous and non-porous substrates. Furthermore, the high stability of these fluorescent developers allowed for preserving developed LFPs for a long time without degradation (Barros et al., 2020). Silica matrices were widely used for fingerprint development, as these silica-based powders had microsized structures as well as good adherence to fingerprint residues. A series of new benzazole fluorescent small molecules were synthesized and used successfully for the visualization of LFPs on the sticky side (Barros and Stefani, 2016), while they could not develop LFPs on any smooth surfaces with lower amount of fingerprint residues. Benzazole dyes were made into the form of microstructured fluorescent powders by a silica matrix, showing excellent fluorescent images for fingerprint detection on different types (porous and non-porous) and colors (dark, white, and multi-colored) of surfaces. Compared with commercially available black, white, and fluorescent powders (Sirchie®), the developed microstructured powders showed intense fluorescence emission in the blue-green region, and a sharp contrast with the fingerprint residues when exposed to 365 nm UV light, producing distinct ridge details on all examined surfaces (Barros and Stefani, 2019).

**Figure 1:**
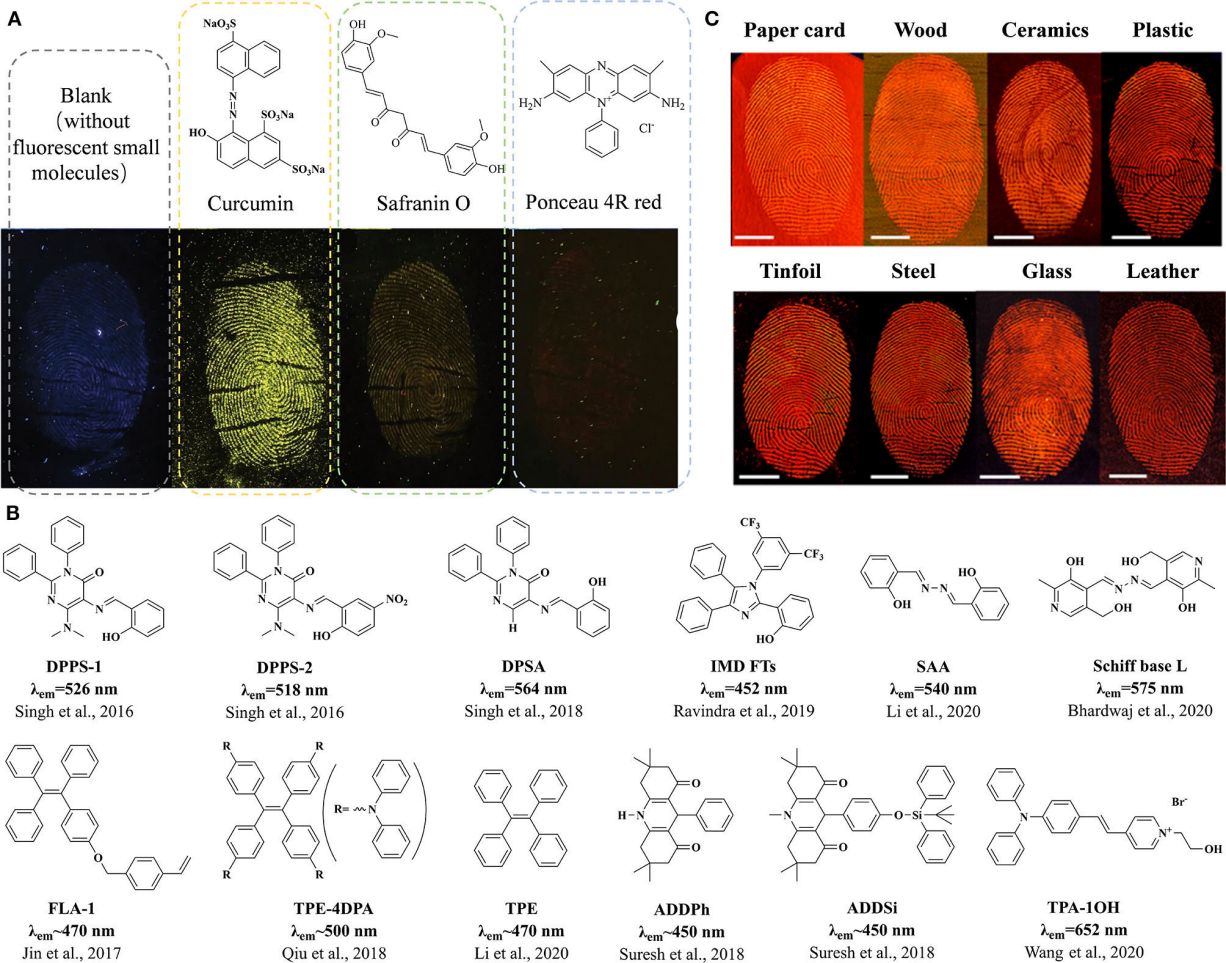
Fluorescent small molecule structures and fluorescent images of LFPs developed by fluorescent small molecule-based materials. **(A)** The structures and fluorescent LFP images of some small molecules incorporated with SiNPs and starch powders. Modified from Rajan et al. (2019), with permission. **(B)** The structures of small molecules with AIE features for LFP imaging. **(C)** Fluorescent LFP images on various surfaces treated by a TPA-1OH aqueous solution. Modified and adapted with permission from Wang et al. (2020). Copyright (2020) American Chemical Society.

As silicon nanoparticles (SiNPs) are widely applied in materials and biomedical fields because of the controllable size, facile synthesis, ease of functionalization, excellent biocompatibility, optical transparency, and low toxicity, SiNP is a good matrix for organic dyes to improve the affinity to fingerprint residues in recent research (Song and He, 2019; Zhu et al., 2019; Abu-Thabit and Ratemi, 2020). Fluorescent SiNPs modified by organic dye Pigment Red 254 were coated with polyvinylpyrrolidone (PVP) to improve their binding affinity to LFPs. The modified organic dye powders were used as a powder-dusting material for effective LFP detection on representing hydrophilic and hydrophobic substrates, resulting in a good definition of enhanced detection with well-defined ridge detail images without background staining (Kim et al., 2016). Twelve fluorescent organic dyes were labeled to SiNPs with tunable sizes, tailored hydrophobicity, and low polydispersity to evaluate the fluorescent performance. Then, three different organic fluorophore powders were selected to be systematically investigated. The hydrophilic fluorophore, butenamine derivative of FITC (WA6), coupled with SiNP powders was found to be an excellent LFP imaging reagent for clear detection of LFPs with the typical features of second-level detail well-defined ridges and tertiary structure sweat pores of the developed fingerprint (Abdelwahab et al., 2018). A silicone fluorescent small-molecule prepared non-conjugated with SiNPs exhibited excellent bright blue-emissive stability in ultraviolet irradiation and a wide pH range. These fluorescent powders demonstrated to be an effective LFP powder on a variety of substrates under a 365-nm UV lamp, such as glass, wood, stainless steel, and a poly(ethylene) film, resulting in clear fluorescent images of the ridges and spaces with high contrast and resolution of all the three levels of LFPs (Zhang et al., 2019). Considering the safety of the environment and the operators, a natural dye curcumin and two food dyes (safranin O and Ponceau 4R red) incorporated with SiNPs and starch powders were investigated by developing LFPs under the same situation (Figure 1A). Images of the developed LFPs under white and UV light with different qualities of the ridge details after applying four LFP powders demonstrated that only curcumin retained good contrast after its application to the fingerprints, and curcumin LFP powders were demonstrated to be effective on difficult and challenging surfaces (Rajan et al., 2019).

### Fluorescent Molecules With AIE Features for LFP Imaging

Fluorescent molecules with AIE features are designed for LFP imaging with high contrast and different colors (Figure 1B), and the development is mainly in wet methods using different solutions. AIE-active diphenylpyrimidinone derivative DPPS-1 (Singh et al., 2016) and DPSA (Singh et al., 2018) with excited-state intramolecular proton transfer (ESIPT) mechanisms in the H_2_O/CH_3_CN mixture showed successful applications in visualization of LFPs with the first-level and second-level details on non-porous metal substrates. Acridinediones (ADDPh and ADDSi) with AIE feature were reported to develop LFPs down to the second-level details on different non-porous substrates by a portable wet method in the THF/water mixture for a 2-min enhancement (Suresh et al., 2018). A novel imidazole derivative (IMD FTs) was designed to imaging LFPs with the third-level details (sweat pores) on various porous/semi-porous/non-porous surfaces under a 365-nm UV light (Ravindra et al., 2019). The Schiff base derivative of the vitamin B_6_ cofactor pyridoxal (L) was applied for LFP imaging in the DMSO/H_2_O mixture over a non-porous glass slide (Bhardwaj et al., 2020). A water-soluble probe TPA-1OH was used for expedient real-time (within 1 min) fluorescent imaging of LFPs. The TPA-1OH aqueous solution exhibited excellent fluorescence imaging performance of LFPs under 405 nm light on a variety of substrates, such as tinfoil, stainless steel, glass, leather, cardboard, wood, ceramics, plastic, walls, bricks, and paper (Figure 1C). The LFP images developed by TPA-1OH were evident and intact enough to allow that the level 1–3 details including nanoscopic details could be displayed and analyzed (Wang et al., 2020). Small molecules with AIE features could also be combined with carriers to develop LFPs using the powder dusting method. A tetraphenylethene (TPE) derivative with multiple diphenylamine (DPA), TPE-4DPA, doped with magnetic powders, was successfully applied for LFP development with the second-level details on various smooth and porous substrates (Qiu et al., 2018). A new AIE-active composite salicylaldehyde azine (SAA) and the other two small molecules mixed with montmorillonite (MMT) were, respectively, applied for computer-assisted visualization of LFPs, achieving fluorescent fingerprint images by visualizing the process of the second-level particulars with high quality on virtually all the smooth substrates. Gray value analysis and pattern recognition analysis based on Matlab were conducted to evaluate the quality of the obtained LFP in comparison with the reference fingerprint obtained by Finger Reader (Li et al., 2020). AIE-active organic materials could bring more emission colors, easy functionalization, and rapid development methods for LFP imaging.

## Fluorescent Organic Polymer-Based Materials For LFPs Imaging

Organic polymer is a kind of emerging materials for fluorescent imaging, such as conjugated oligomers, polymer dots, and other various polymeric matrices. Semiconducting polymer dots have recently been proven as a novel type of ultrabright fluorescent probes that can be extensively used in analytical detection. Ninhydrin embedded in the near-infrared fluorescent polymer dot matrix showed the colorimetric and fluorescent dual-readout abilities to detect LFPs on both porous and non-porous surfaces. The developed fingerprints by the dual-readout method clearly revealed level 1–3 details with high contrast, high selectivity, and low background interference (Chen et al., 2016).

**Figure 2:**
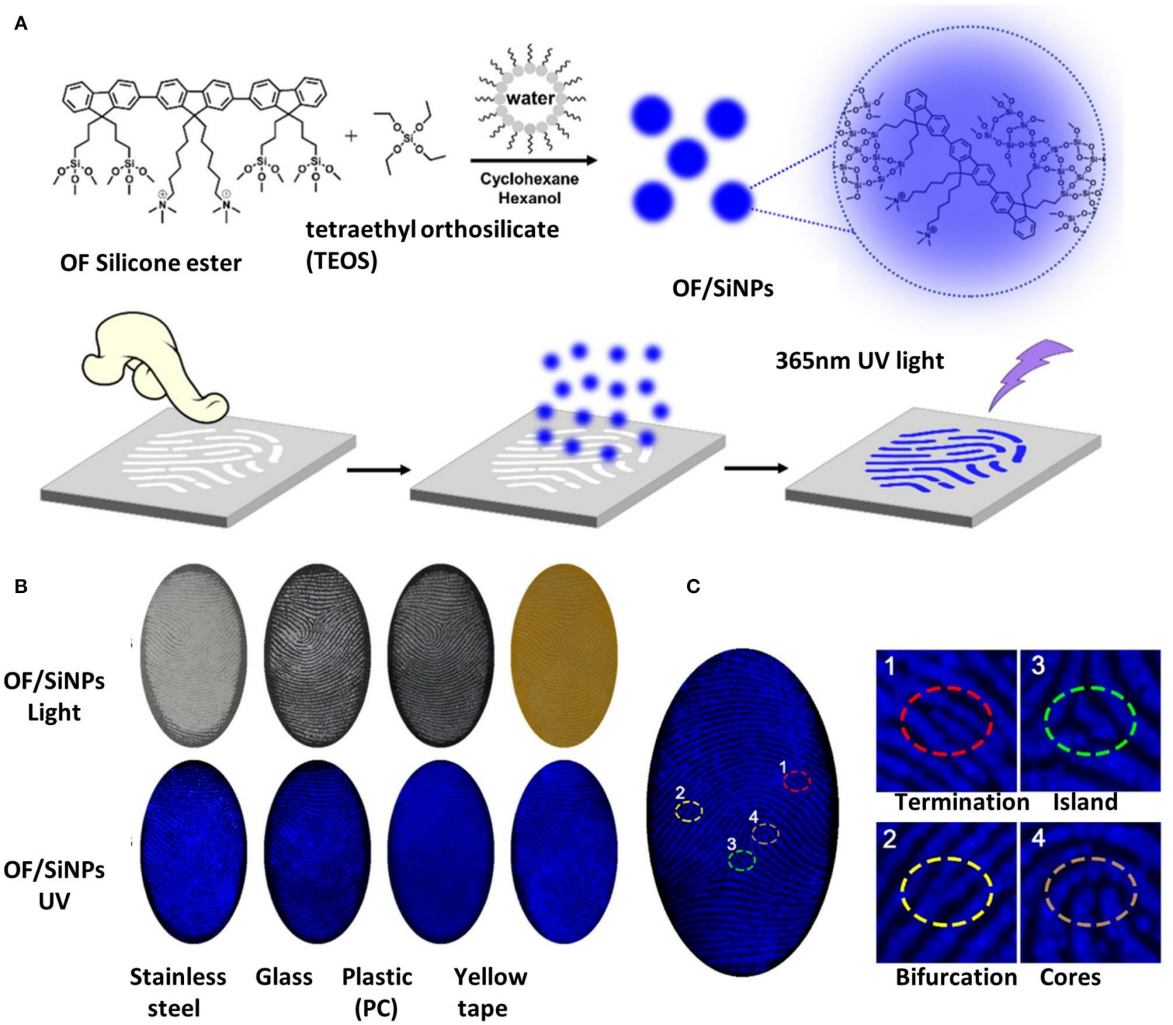
The process and images of LFP development using OF/SiNP powders. **(A)** The synthesis and LFP development process of the fingerprint reagent OF/SiNPs; **(B)** images of developed LFPs on various substrates under room light and 365 nm UV light; **(C)** fluorescence images of latent fingerprints developed on yellow tape, and magnified regional images with specific features, including a termination 1, an island 2, a bifurcation 3, and a core 4. Modified and adapted with permission from Zhang S. et al. (2017). Copyright (2017) American Chemical Society.

Fluorescent powders made of a conjugated oligomer with SiNPs were also applied for LFP imaging. Blue-emissive conjugated oligomers covalently blending to SiNPs with the emission quantum yields as high as 62.2% were used as fingerprint powders on various substrates, such as stainless steel, glass, plastic (polycarbonate, PC), and yellow tapes. Fluorescent images of LFPs on different substrates exhibited clear blue fingerprint patterns under 365-nm UV light, maintaining sufficiently high resolution and contrast between the ridges and spaces, as seen in Figure 2 (Zhang S. et al., 2017). Red-emissive conjugated oligomer with the carrier epoxy-SiNPs allowed clear imaging of LFPs under 365-nm UV light on different kinds of surface, such as smooth or rough surface, with or without background fluorescence, using the powder dusting method. The introduction of epoxy groups on SiNPs could largely enhance the affinity toward fingerprints, compared to that with hydroxy or amino groups. The fluorescent images of LFPs with a clear ridge pattern and high contrast between ridges and furrows proved the promising imaging ability for LFPs, giving detailed both the second-level and third-level features and abundant information (Yang et al., 2019).

Aggregation-induced enhanced emission-active conjugated polyelectrolyte was confirmed the ability for LFP imaging with high resolution on multiple smooth non-porous substrates without any additional treatment. The LFP development process was accomplished simply by immersing fingerprint-loaded substrate into the solution for ~1 min, followed by shaking off the residual polymer solution and air drying. The images revealed clearly the third-level details with high selectivity and high contrast (Malik et al., 2017). A bilayer system based on conjugated and fluorescent polymers was used for the LFP development of stainless steel, with the first layer of polypyrrole or PEDOY electrodeposited onto the surface and the second layer of a fluorescent Poly(2,2′:5′,2″-terthiophene) electrodeposited onto the first layer. This bilayer system could provide images with high definition of the first-level and second-level details in both visible and UV light for personal identification (Costa et al., 2020b).

## Conclusions and Future Perspectives

Given the value of fingerprint evidence in criminal investigations, the main thrust of this mini-review is to remain focused on increasing the likelihood of LFP development based on fluorescent organic materials. Despite traditional LFP powders and fuming methods as standard processes for the development of LFPs in forensic practice for years, there has been significant research to discover new fluorescent imaging methods that offer operational advantages. As LFP development is daily work of crime science investigators and forensic scientists that may perform everywhere, LFP-developing reagents should be friendly to the environment and users. Fluorescent organic materials with various improvers possess excellent biocompatibility, low or no cytotoxicity, low cost, convenience, and environment friendliness, showing excellent performance as LFP development reagents with less dependency on instruments, high contrast, and room operating temperature in the mentioned research. As fluorescent organic materials applied for LFP imaging, the handing of LFPs can be in powder dusting or wet methods, which are common in standard processes for the development of LFPs in forensic practice, making the new technology easy to accept, and promote.

Despite the remarkable applicability and performances of various fluorescent organic materials, there are still challenges that need to be addressed. First, most of the research focus on the application of several LFP substrates. The ideal LFP developing method should be useful on both porous and non-porous, both dry and wet surfaces, so more LFP substrates should be discussed in the comprehensive evaluation of a new method.

The fluorescent imaging of LFPs with low background has obvious advantages of sensitivity and convenience over traditional LFP development, achieving LFP images with high contrast and resolution. Taking the three levels of features into consideration, most of the research reached the second-level or third-level precision with the potential of further practical applications, even validated by the scientific protocol of the Police Automated Fingerprint Identification System or computer-assisted evaluation programs. Moreover, smartphones and a specific designed digital-processing program for imaging enhancement are applied for the analysis of the LFP imaging results. In practice, fingerprints from crime scene are always incomplete or damaged and need more details of third-level precision to be recognized. Fluorescent imaging methods with third-level precision or imaging enhancement are of great practical significance. Meanwhile, the conditions of the research were ideal, and some reported works talked about aged LFPs, but overlapped LFPs, immersed LFPs, and DNA-recovered LFPs were barely mentioned. Finally, antibodies, aptamers, or molecular imprinting materials combined with QDs or rare earth nanomaterials were successfully applied for fingerprint residues in fingerprints, such as illegal drugs and explosives, but these highly selective reagents have not been combined with fluorescent organic materials (Lam et al., 2016; Zhao et al., 2016; Liu et al., 2018a,b; Zhou et al., 2019). Along with new reagents introduced in the field, the fluorescence quantum yield and affinity to fingerprint residues of fluorescent organic materials should be further optimized to improve the contrast of LFP imaging. On an operational level, there is still a requirement for more research on rapid, selective, different-colored, and convenient fluorescent imaging methods for imperfect LFPs with higher third-level precision on different types of substrates.

Continued research in this field will require expertise in chemical synthesis, material science, life science, and advanced spectroscopy, and thus, there is ample room for forensic science researchers to help improve and extend the key forensic technique. Active research in this area will continue into the foreseeable future in order to promote and improve latent fingerprint development to assist with the efficiency of criminal investigations and personal identifications.

## Author Contributions

JL and FM conceived the concept, conducted literature survey, drafted, and revised the manuscript. All the authors organized figures and approved it for publication.

## Conflict of Interest

The authors declare that the research was conducted in the absence of any commercial or financial relationships that could be construed as a potential conflict of interest.
